# Supplementary material for: Reactive oxygen species affect the potential for mineralization processes in permeable intertidal flats
Source: Nat Commun. 2023 Feb 20;14:938. doi: 10.1038/s41467-023-35818-4 (PMC9941506; doi:10.1038/s41467-023-35818-4)
Supplement: Supplementary file 1 — Supplementary Information [file 41467_2023_35818_MOESM1_ESM.pdf]

Supplementary Information

Reactive oxygen species affect the potential for mineralization processes in permeable intertidal flats

Marit R. van Erk[1,3,#,*], Olivia M. Bourceau[1,#,*], Chyrene Moncada[1], Subhajit Basu[1,4], Colleen M. Hansel[2], Dirk de Beer[1]

[1]Max Planck Institute for Marine Microbiology, Bremen, Germany

[2]Department of Marine Chemistry and Geochemistry, Woods Hole Oceanographic Institution, Woods Hole, Massachusetts, USA

[3]Present address: Department of Earth Sciences, Utrecht University, Utrecht, The Netherlands

[4]Present address: School of Health Sciences and Technology (SoHST), University of Petroleum and Energy Studies (UPES), Dehradun, Uttarakhand 248007, India

[#] *These authors contributed equally*

* Corresponding authors: Marit R. van Erk (merk@mpi-bremen.de), Olivia M. Bourceau (obourcea@mpi-bremen.de)

Content

1. Supplementary Figure 1 to 13

2. Supplementary Table 1 to 7

3. Supplementary Information hydrogen peroxide sensor

4. Supplementary References

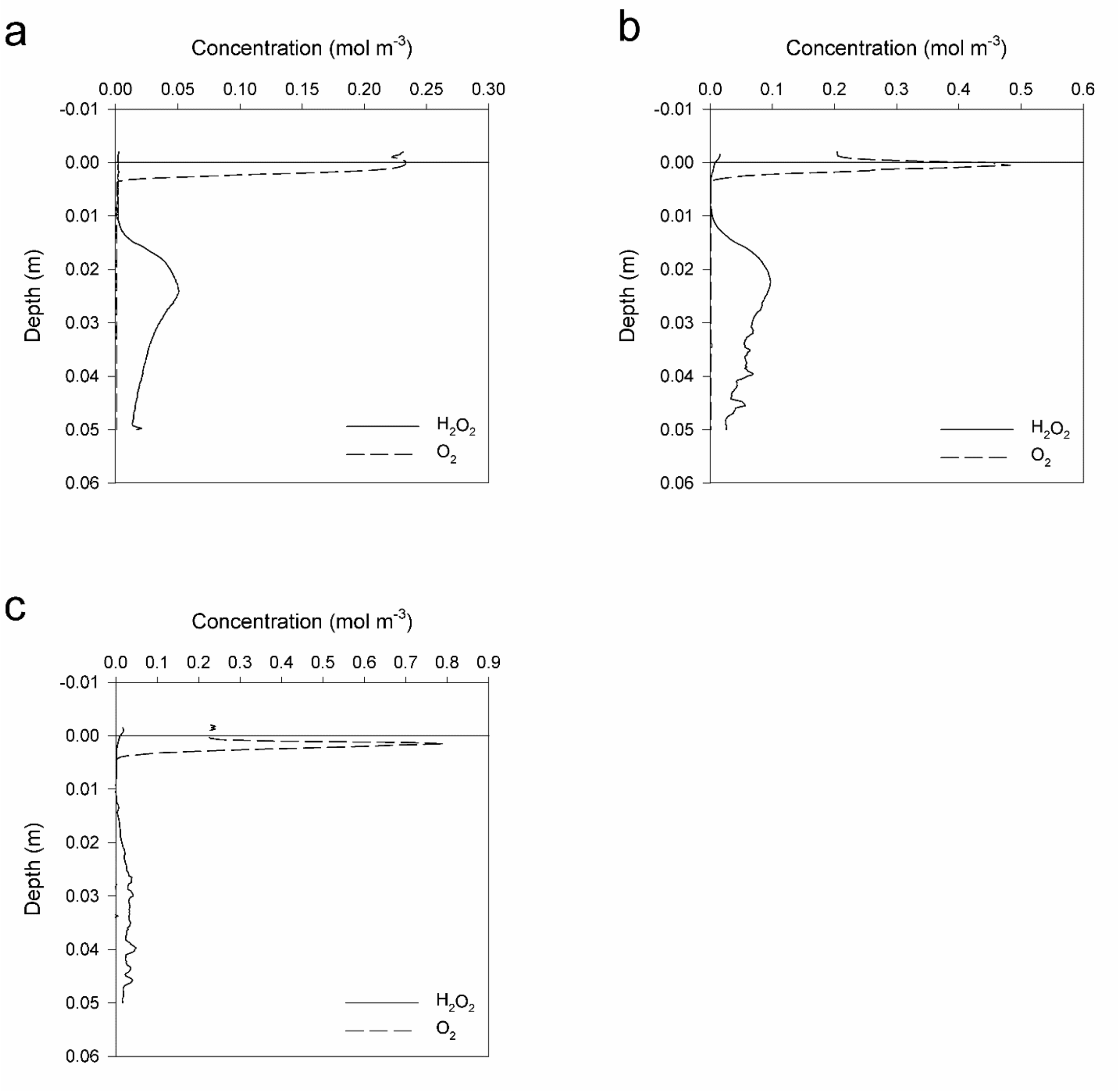

Supplementary Figure 1: Steady-state microprofiles of hydrogen peroxide (H_2O_2; solid line) and oxygen (O_2; dashed line) measured in a sediment core. Microprofiles are in the same sediment core as in Fig. 1a,b, but measured a day later. **a:** In the dark, **b** and **c**: In the light. a and b are measured 1 hr 45 min apart. b and c are measured 45 min apart.

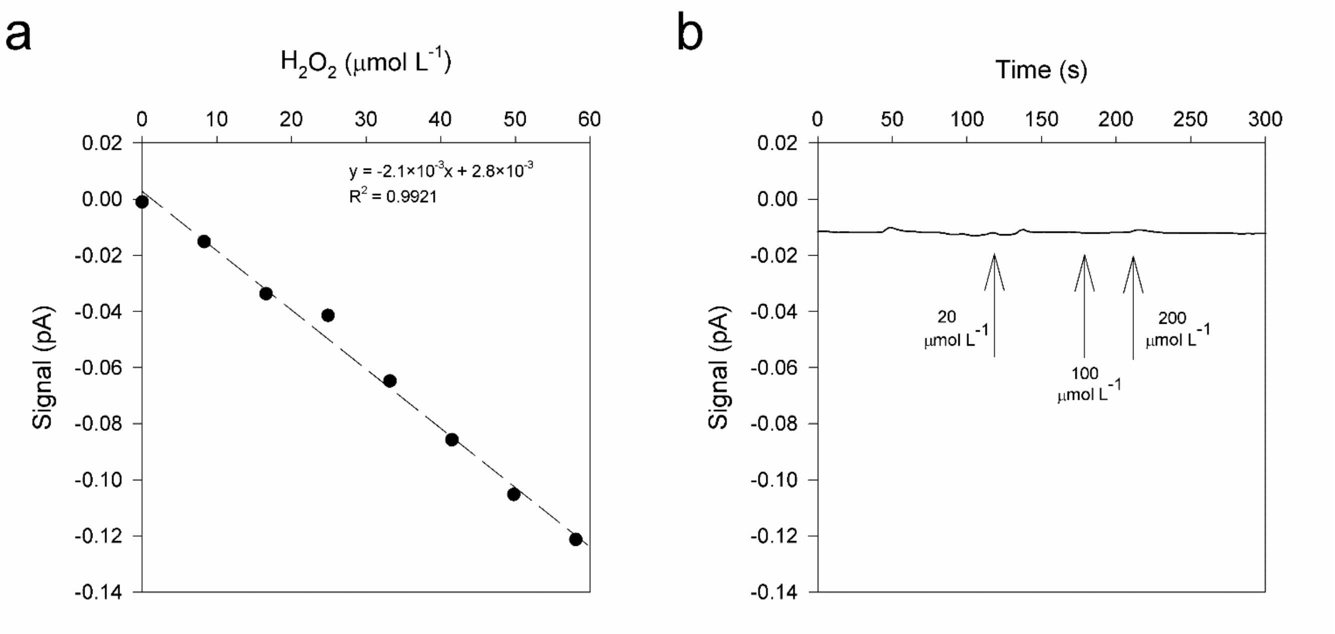

Supplementary Figure 2: a: Calibration for a hydrogen peroxide (H_2O_2) sensor with ferrozine in the electrolyte (50 µmol L^{-1}), **b:** Signal over time for the same sensor as in Supplementary Fig. 2a. Fe^{2+} was added step-wise to a 500 mL solution of N_2-flushed seawater of pH 3. Arrows represent the time at which Fe^{2+} was added, with the tested concentration of Fe^{2+}.

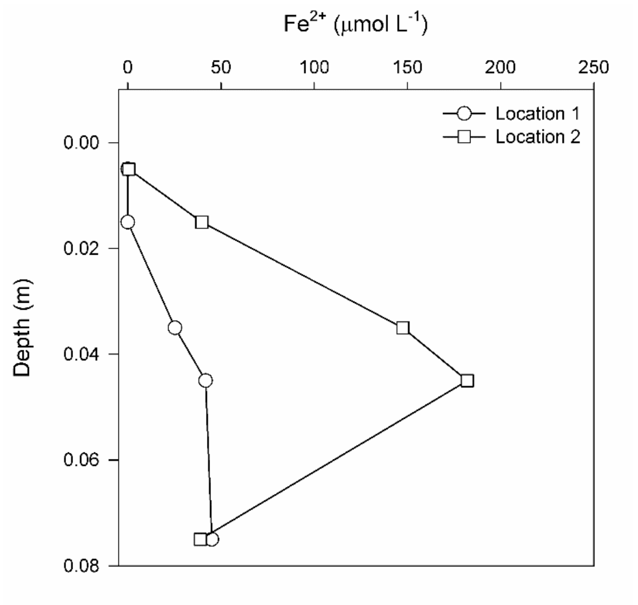

Supplementary Figure 3: Dissolved ferrous iron (Fe^{2+}) concentrations measured at two locations. Porewater was collected in situ using Rhizons, and directly fixed in the attached syringe using ferrozine. A subsample of the porewater was used for the chemiluminescence measurements of Supplementary Fig. 5b.

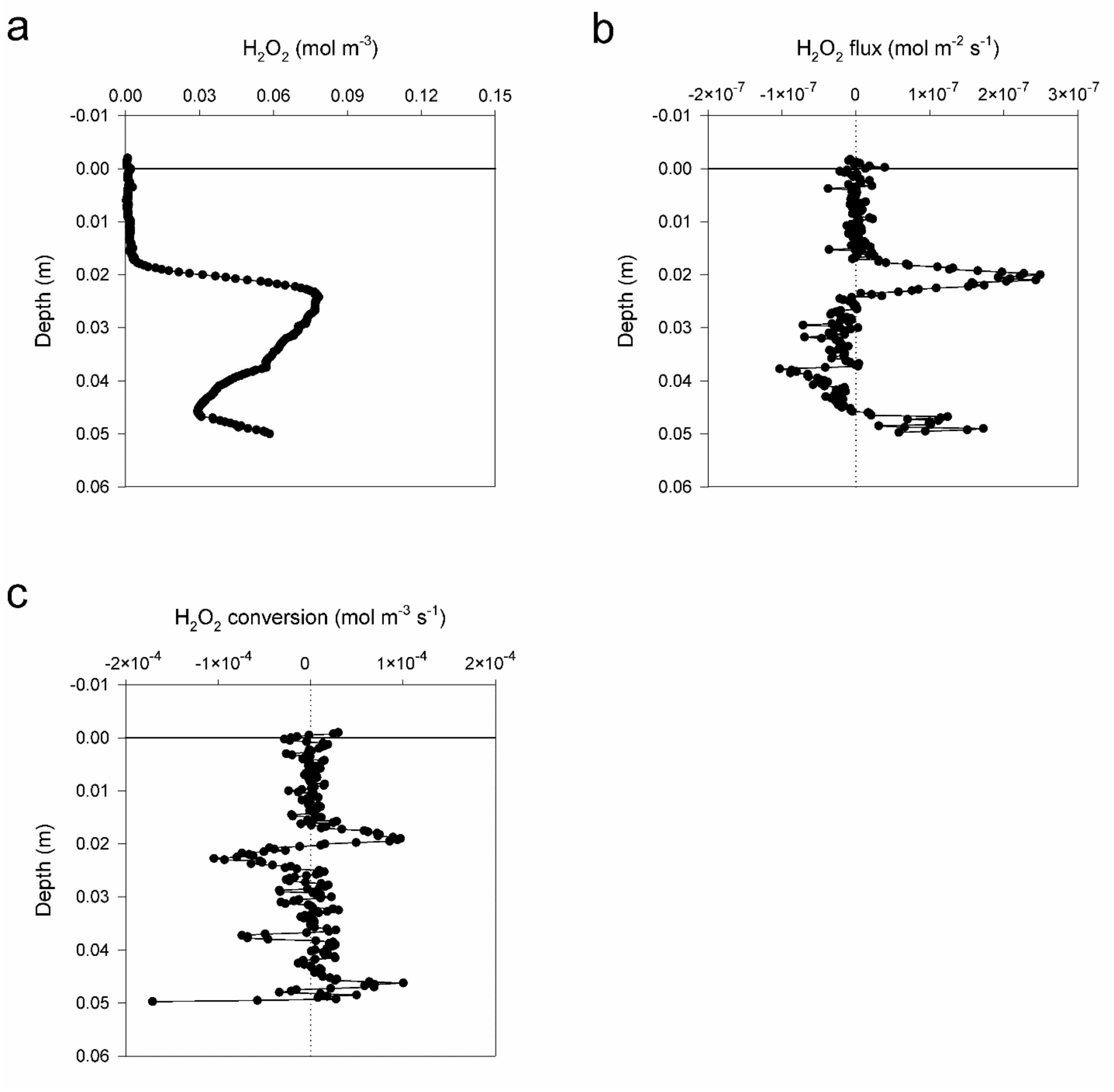

Supplementary Figure 4: Concentrations, fluxes and conversion rates of hydrogen peroxide (H_2O_2) in a sediment core. All are from the same sediment core, collected March 18th 2021. **a:** Steady-state microprofile of hydrogen peroxide, the same microprofile as shown in Fig. 1b, **b:** hydrogen peroxide, calculated from the steady-state microprofile of Supplementary Fig. 4a. Positive values indicate upward fluxes, negative values downward fluxes, **c:** hydrogen peroxide conversion within a sediment core, calculated from the hydrogen peroxide fluxes of Supplementary Fig. 4b. Positive values indicate production, negative values consumption.

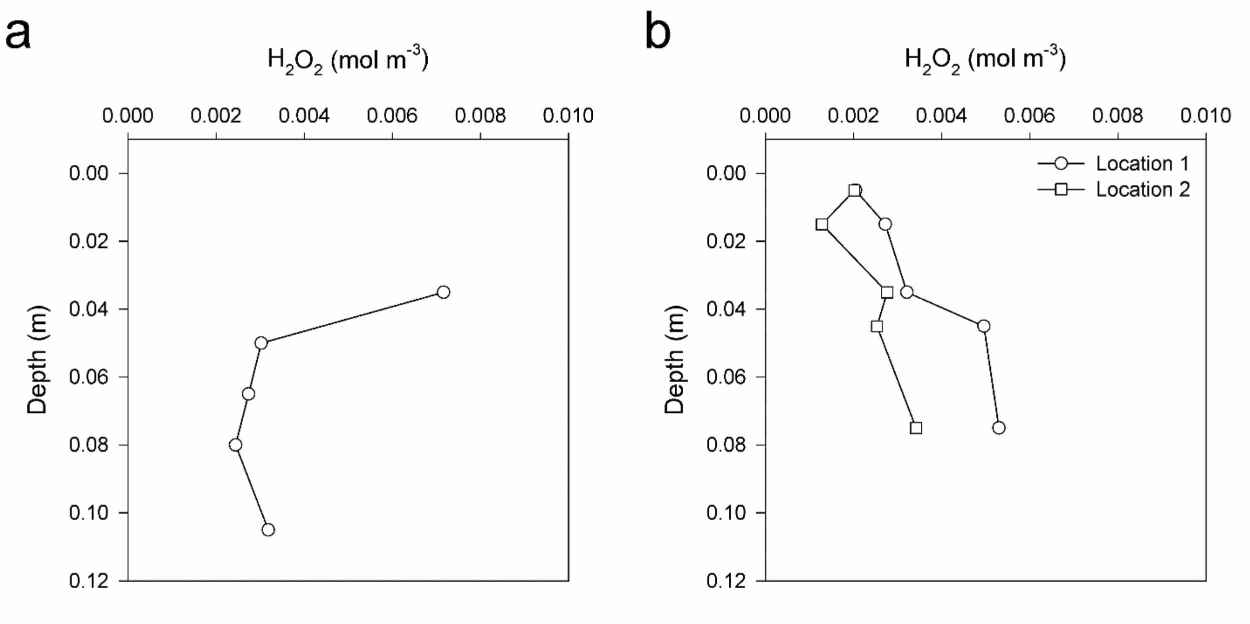

Supplementary Figure 5: Hydrogen peroxide (H_2O_2) concentrations in intertidal permeable sediment. **a:** Hydrogen peroxide concentrations measured in porewater from a sediment core using a chemiluminescent technique. No measurements were conducted above 4 cm depth, due to an absence of openings in the core through which sampling with Rhizons occurred in this depth interval, **b:** Hydrogen peroxide concentrations measured in porewater extracted with Rhizons on the flat from 2 locations using a chemiluminescent technique.

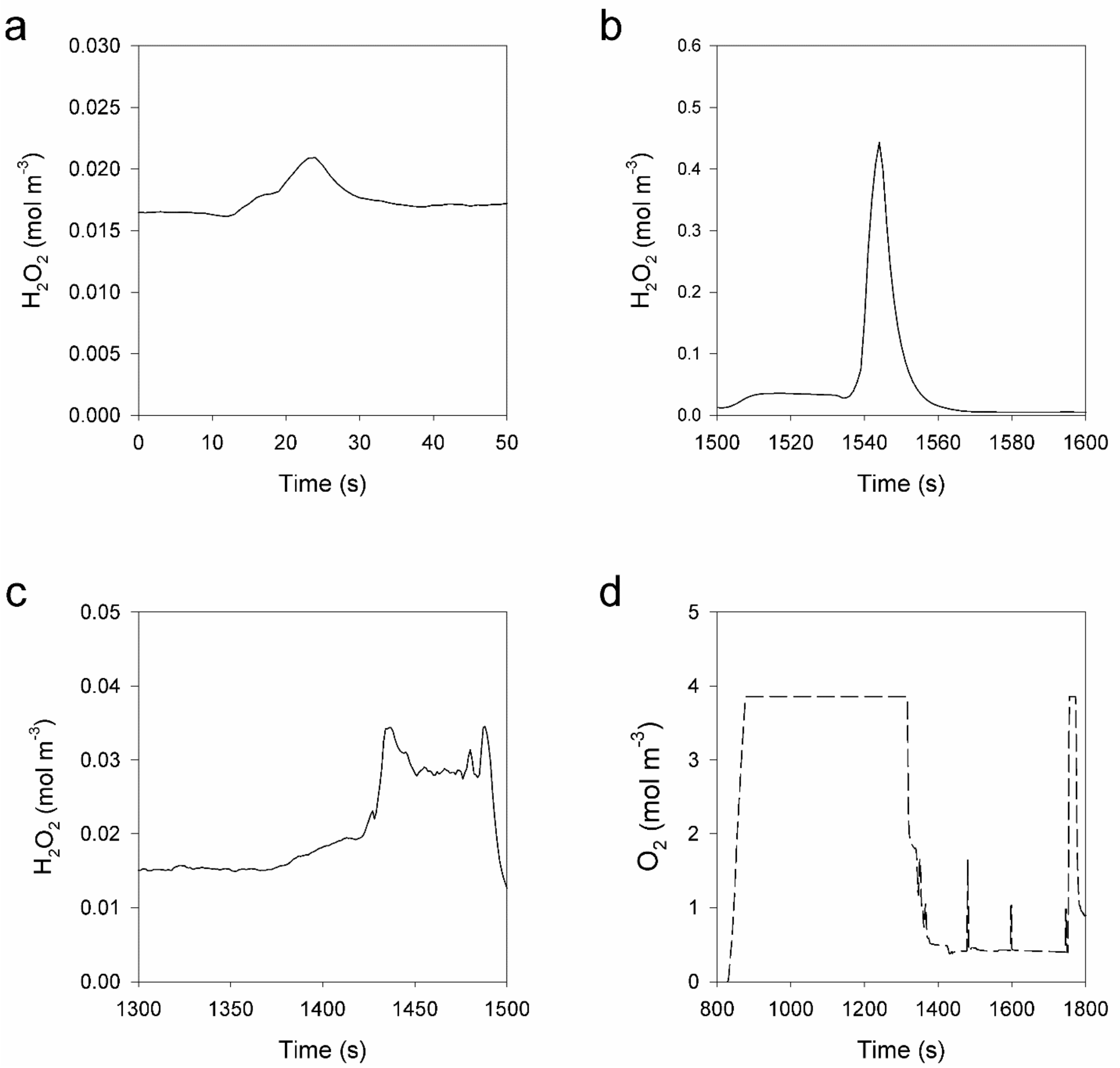

Supplementary Figure 6: Dynamics of hydrogen peroxide (H_2O_2; solid line) and oxygen (O_2; dashed line) in a sediment core. Microsensors were at a stable position (3 cm depth). **a** and **d** are from a parallel core to that of Fig. 2a-c. **a:** Hydrogen peroxide concentrations after injection of oxygenated seawater close to the hydrogen peroxide sensor. Seawater was injected at t=0. **b** and **c:** Transient peaks of hydrogen peroxide after injection of hydrogen peroxide close to the hydrogen peroxide sensor. **d:** Oxygen concentrations after several injections of hydrogen peroxide close to the oxygen sensor. Above around 4 mol m^{-3}, the oxygen sensor was oversaturated.

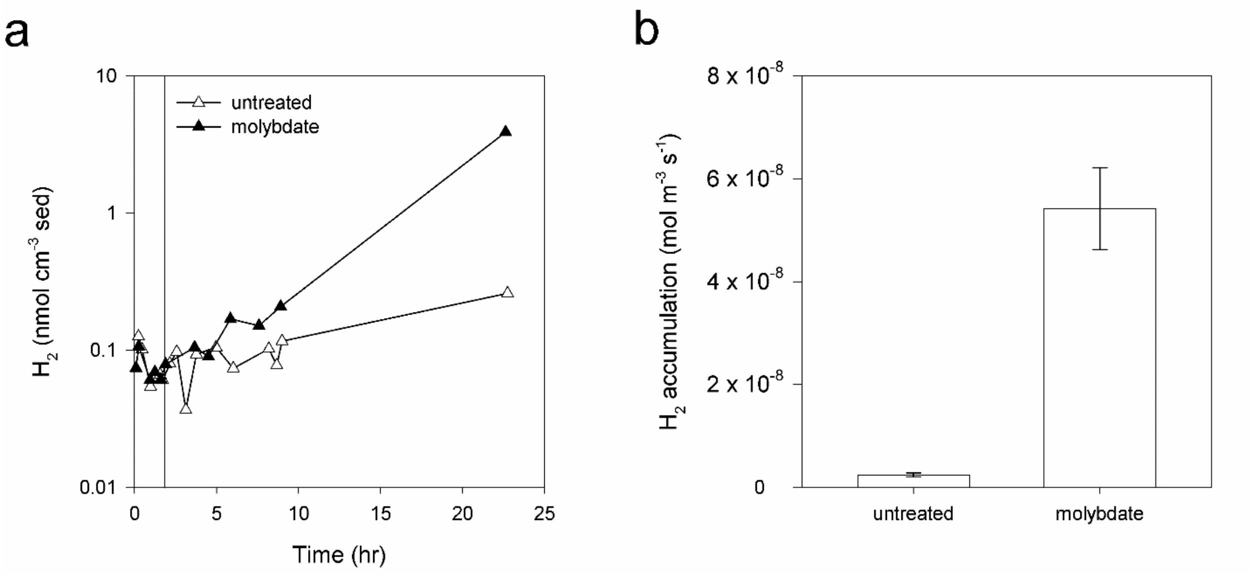

Supplementary Figure 7: Hydrogen (H_2) accumulation. **a:** Hydrogen concentrations in slurries over an oxic-anoxic transition with (closed triangles) and without (open triangles) molybdate, an inhibitor for sulfate reduction, plotted against a log scale. Slurries were from sediment collected May 25th, 2020. The vertical line indicates the point where slurries became anoxic, which was the same for both untreated and molybdate-treated slurries, **b:** Hydrogen accumulation after slurries turned anoxic for slurries with and without (untreated) molybdate. Hydrogen accumulation was calculated from the anoxic period of Supplementary Fig 7a. Error bar represents standard error of the linear trend line.

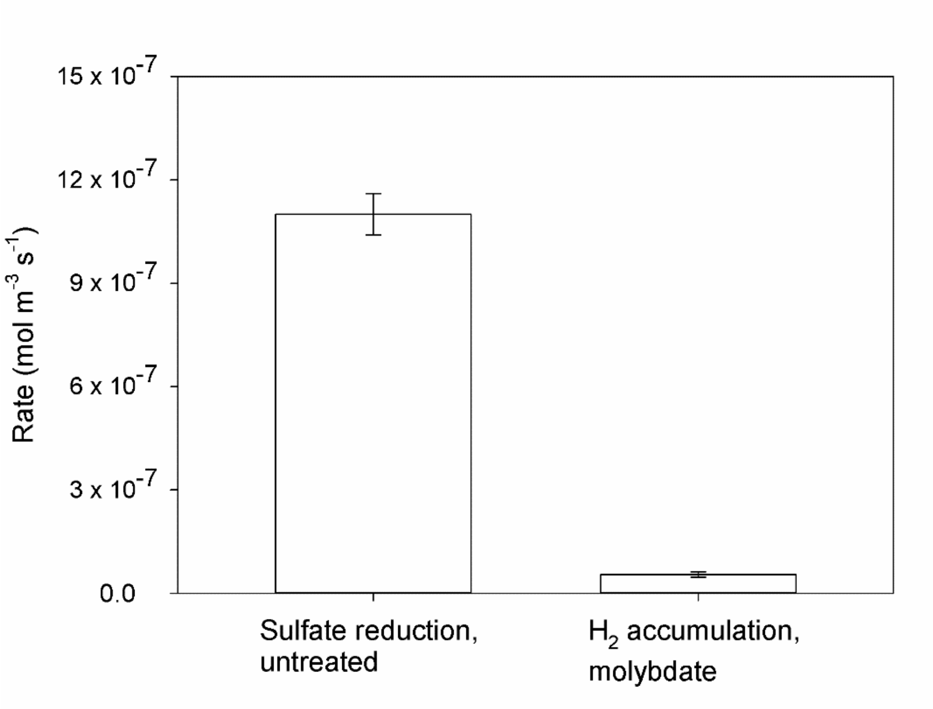

Supplementary Figure 8: Rates of sulfate reduction and hydrogen (H_2) accumulation for the anoxic period. The sulfate reduction rate was calculated from the anoxic period of Supplementary Fig. 9a. The hydrogen accumulation rate was calculated from the anoxic period of Supplementary Fig. 7a. Error bars represent standard error of the linear trend line.

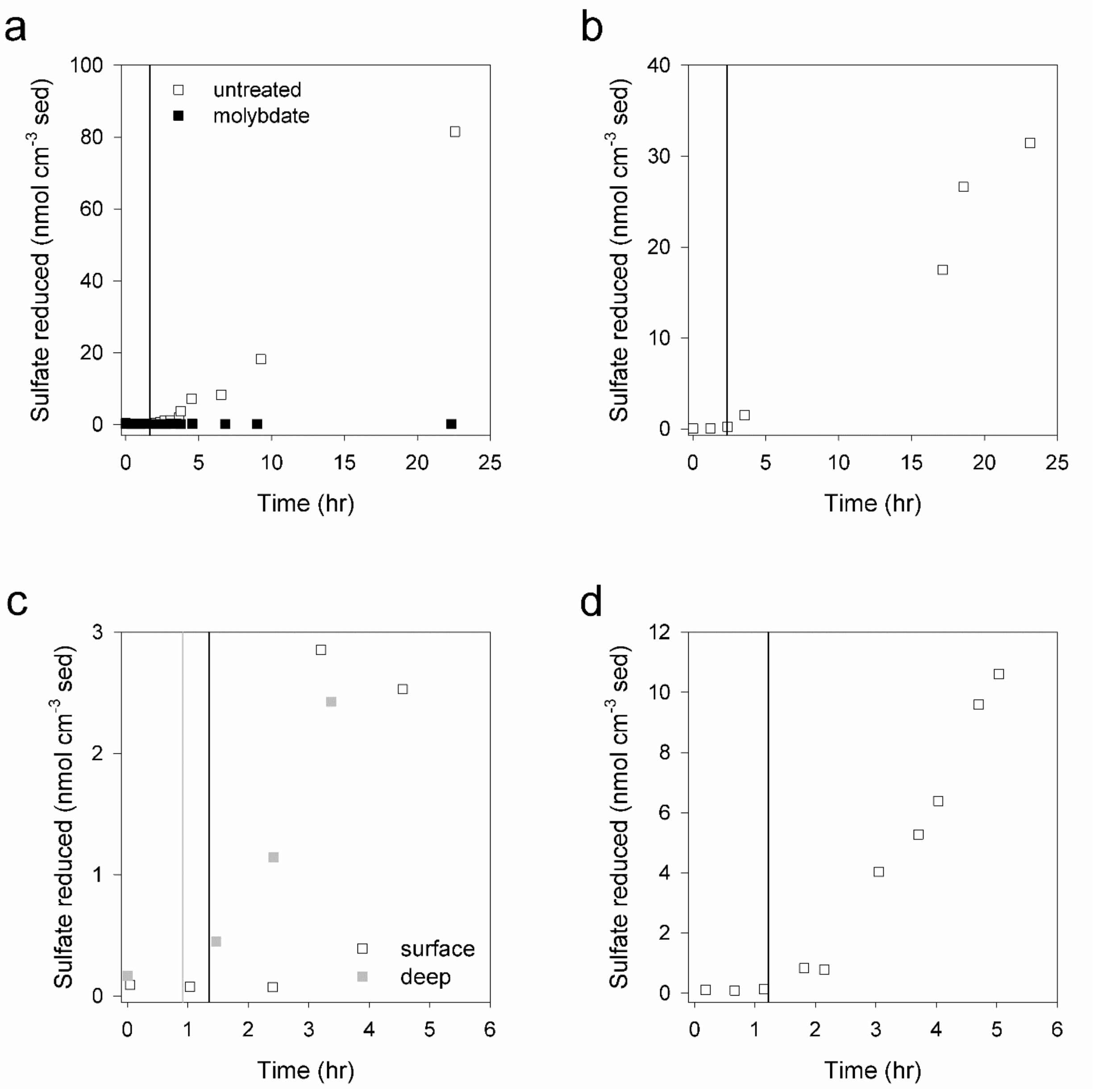

Supplementary Figure 9: Reduced sulfate in slurries over an oxic-anoxic transition. The vertical lines indicate the transition from oxic to anoxic conditions. **a:** Slurries from surface sediments (open squares) and surface sediments treated with molybdate (closed squares), collected May 25th. Untreated and molybdate-treated slurries became anoxic at the same time, **b:** Slurries from surface sediments collected June 15th, **c:** Slurries from surface (0 - 2 cm depth; open squares) and deep (10 - 14 cm depth; grey squares) sediments collected July 28th. The black vertical line represents the transition to anoxic conditions for surface sediments, the grey line that for deep sediments, **d:** Slurries from surface sediments collected October 8th.

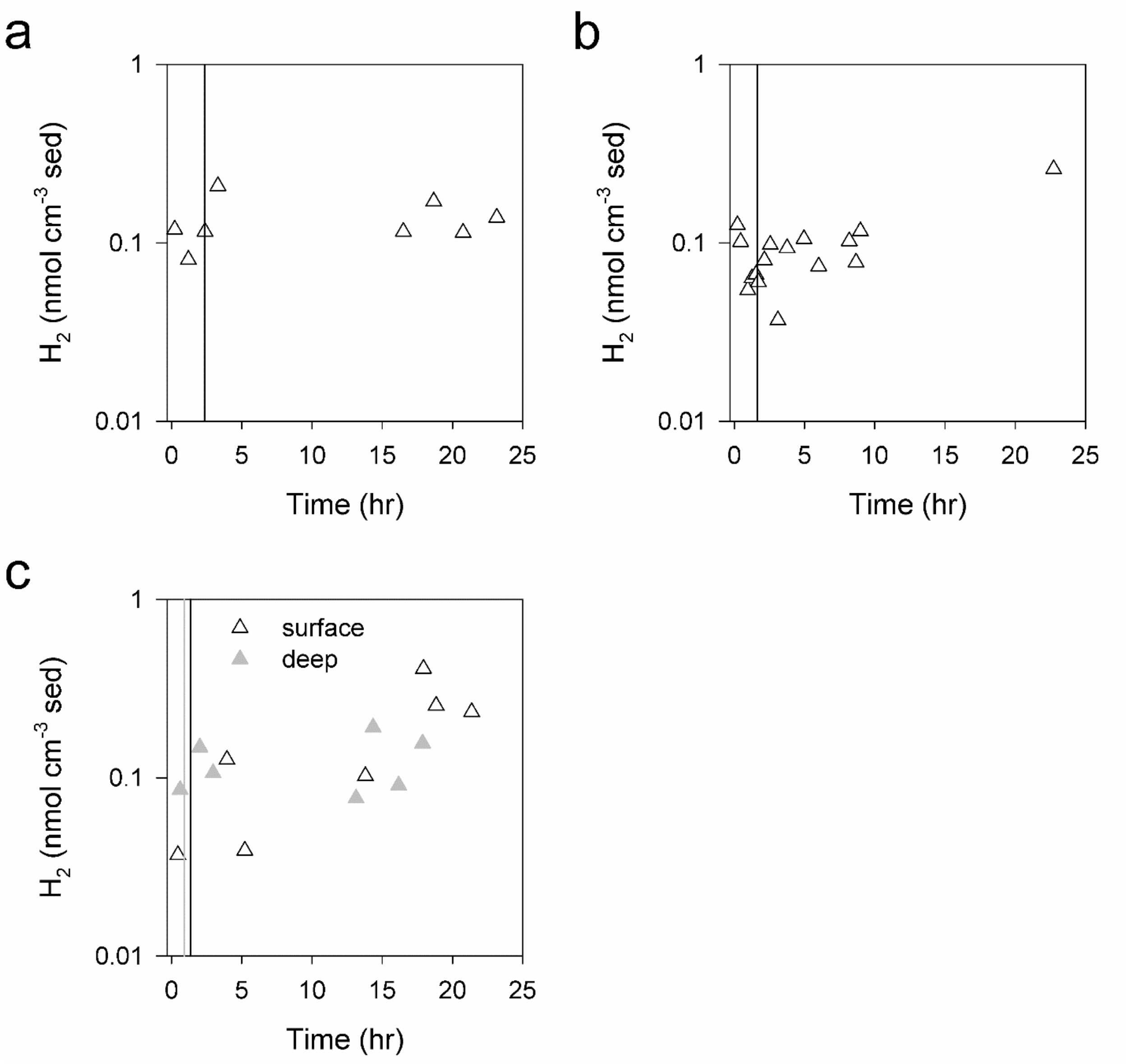

Supplementary Figure 10: Hydrogen (H_2) concentrations in slurries over an oxic-anoxic transition. The vertical lines indicate the transition from oxic to anoxic conditions, **a:** Hydrogen concentrations for sediments collected June 15th, **b:** Hydrogen concentrations for sediments collected May 25th, **c:** Hydrogen concentrations in slurries for surface (0 - 2 cm depth; open trianges) and deep (10 - 14 cm depth; grey triangles) sediments collected July 28th. The black vertical line represents the transition to anoxic conditions for surface sediments, the grey line that for deep sediments.

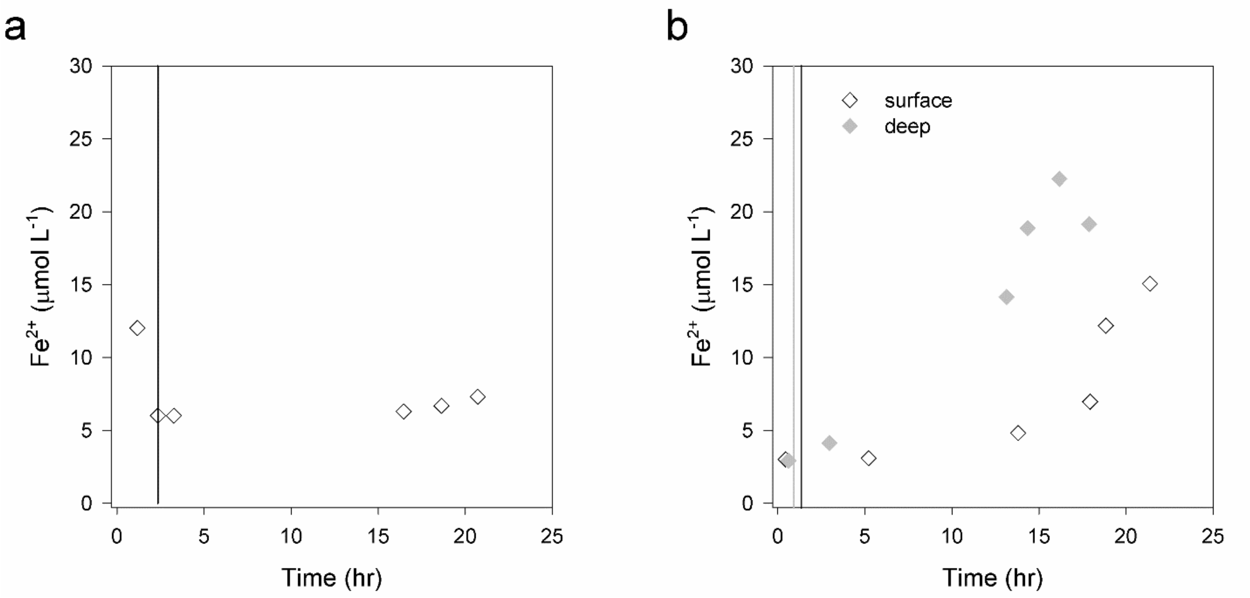

Supplementary Figure 11: Porewater ferrous iron (Fe^{2+}) concentrations in slurries over an oxic-anoxic transition. The vertical lines indicate the transition from oxic to anoxic conditions, **a:** Slurries from surface sediments collected June 15th, **b:** Slurries from surface (0 – 2 cm depth; open diamonds) and deep (10 – 14 cm depth; grey diamonds) sediments collected July 28th. The black vertical line is the transition to anoxic conditions for surface sediments, the grey line for deep sediments.

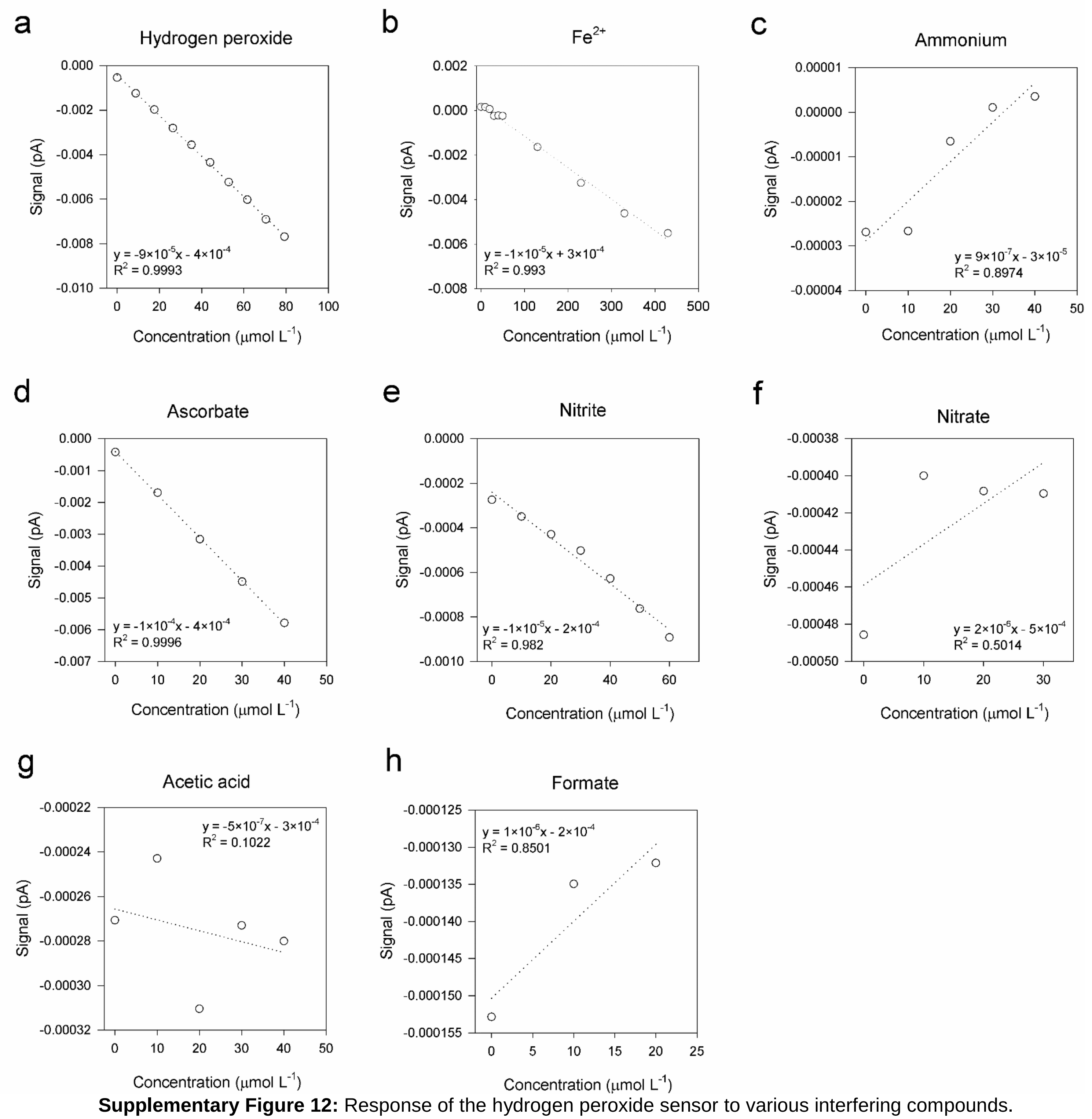

Supplementary Figure 12: Response of the hydrogen peroxide sensor to various interfering compounds. **a:** Hydrogen peroxide, **b:** Ferrous iron (Fe^{2+}), **c:** Ammonium, **d:** Ascorbate, **e:** Nitrite, **f:** Nitrate, **g:** Acetic acid, **h:** Formate.

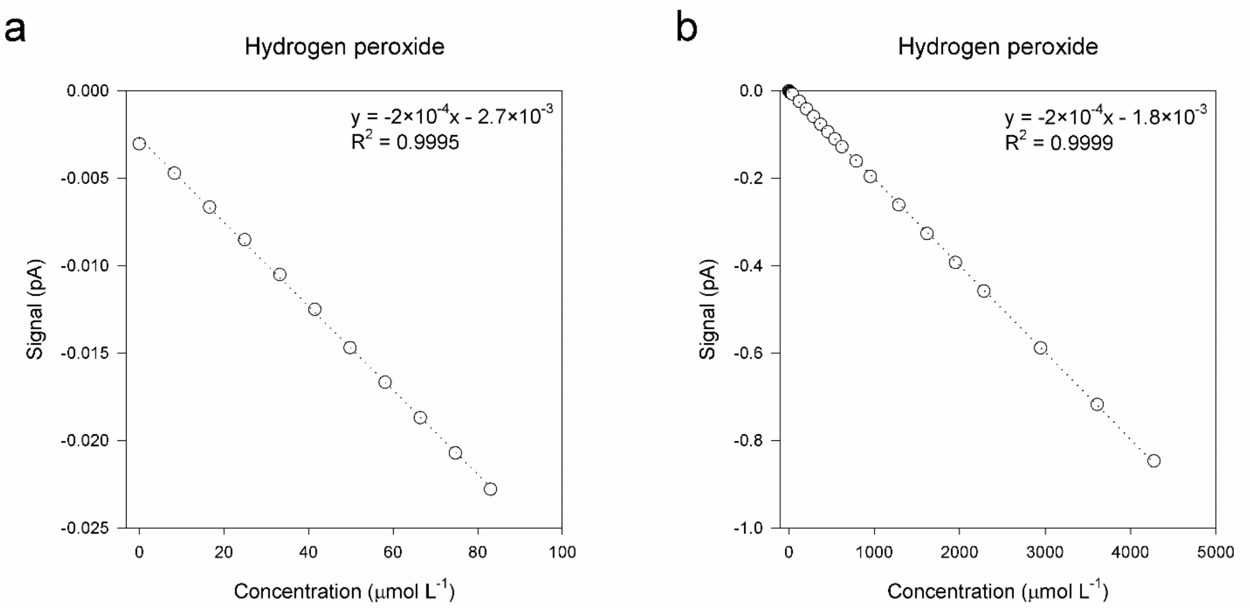

Supplementary Figure 13: Examples of calibrations of the hydrogen peroxide sensor with ferrozine in the electrolyte. **a:** for a lower, and **b:** for a higher hydrogen peroxide concentration range.

Supplementary Table 1: Sedimentary total iron (Fe) concentrations in surface sediment (0-2 cm depth) for sediments collected in May and July 2020, and in deep sediment (10-14 cm depth) for sediments collected in July 2020.

**Sample**	**Fe (µmol g^{-1} sed)**
Surface, May 2020	10.41
Surface, May 2020	7.66
Surface, May 2020	9.31
Surface, May 2020	8.38
Surface, May 2020	8.85
Surface, May 2020	9.94
Surface, May 2020	7.63
Surface, May 2020	11.32
Surface, May 2020	10.31
Surface, May 2020	13.86
Surface, July 2020	1.89
Surface, July 2020	1.70
Surface, July 2020	2.30
Surface, July 2020	2.14
Surface, July 2020	3.53
Deep, July 2020	3.92
Deep, July 2020	3.79
Deep, July 2020	3.71
Deep, July 2020	3.94
Deep, July 2020	4.47

Supplementary Table 2: Carbon turnover in untreated slurries and slurries treated with a combination of catalase and superoxide dismutase (CAT+SOD). Sediments were collected June 15th.

Treatment	Oxygen consumption rate (mol C m^{-3} s^{-1})	Sulfate reduction rate (mol C m^{-3} s^{-1})	Total turnover (mol C m^{-3} s^{-1})
Untreated	4.79×10^{-5}	8.28×10^{-7}	4.87×10^{-5}
CAT+SOD	1.93×10^{-4}	1.39×10^{-6}	1.94×10^{-4}

Supplementary Table 3: Hydrogen (H_2) concentrations from sediment cores, collected July 2020 and March 2021.

Core number	Depth (cm)	H_2 (nmol cm^{-3} sed)
1, July 2020	0	0.047
1, July 2020	2	0.031
1, July 2020	4	0.016
1, July 2020	6	0.029
1, July 2020	8	0.030
1, July 2020	10	0.018
2, July 2020	0	0.039
2, July 2020	2	0.028
2, July 2020	4	0
2, July 2020	6	0.049
2, July 2020	8	0.063
2, July 2020	10	0.029
3. July 2020	0	0.029
3. July 2020	2	0.011
3. July 2020	4	0.034
3. July 2020	6	0.038
3. July 2020	8	0.019
3. July 2020	10	0.029
1, March 2021	0	0.047
1, March 2021	2	0.035
1, March 2021	4	0.031
1, March 2021	6	0.036
1, March 2021	8	0.048
1, March 2021	10	0.040
1, March 2021	12	0.051
1, March 2021	14	0.031
2, March 2021	0	0.046
2, March 2021	2	0.041
2, March 2021	4	0.054
2, March 2021	6	0.041
2, March 2021	8	0.040
2, March 2021	10	0.047
2, March 2021	12	0.051
2, March 2021	14	0.040
2, March 2021	16	0.042

Supplementary Table 4: Porewater sulfide concentrations from cores collected May 2020.

Location	Depth (cm)	Sulfide (μmol L^{-1})
1	2	0.00
1	3	0.00
1	5	0.00
1	7	0.48
1	9	0.48
1	11	0.56
2	1	0.56
2	3	0.15
2	5	0.31
2	7	0.00
2	9	1.38

Supplementary Table 5: Methane concentrations in untreated surface sediment slurries, from sediments collected July 2020.

Time (hr)	Methane (nmol cm^{-3} sed)
1.48	1.08
4.42	1.62
6.03	1.34
13.48	1.46
17.75	1.59
20.45	1.32
21.83	1.65

Supplementary Table 6: Sulfide concentrations in untreated surface sediment slurries, from sediments collected May 2020.

Time (hr)	Sulfide (μmol L^{-1})
0.45	1.44
5.22	0.45
13.80	1.02
17.93	1.96
18.83	0.90
21.37	2.41

Supplementary Table 7: Interference of various compounds with the hydrogen peroxide microsensor. Interference (in %) is calculated as: sensitivity/H_2O_2 sensitivity × 100.

	**Tested concentration range (μM)**	**Sensitivity (pA μM^{-1})**	**Interference (%)**
Ammonium	0 - 40	9×10^{-7}	1
Nitrite	0 - 60	1×10^{-5}	11
Nitrate	0 - 30	2×10^{-6}	2
Formate	0 - 20	1×10^{-6}	1
Acetic acid	0 - 40	5×10^{-7}	0.6
Ascorbate	0 - 40	1×10^{-4}	111
Fe^{2+}	0 - 430	1×10^{-5}	11
Fe^{2+} (sensor with ferrozine)		none	

Supplementary Information hydrogen peroxide sensor

The sensor consisted of an etched 50 µm-thick platinum anode plated with platinum chloride (8% $PtCl_4$ in MilliQ water), an etched 100 µm-thick platinum guard, and a thick platinum reference. The anode, guard and reference were mounted in a glass casing, with the sensing anode at a distance of ca 50 µm from the tip. The tip of the outer capillary had a diameter of 25 - 30 µm and a tip opening of 10 µm. Before mounting the electrodes, the tip of the outer capillary was sealed by a thin polyurethane membrane (D6)[1]. The membrane was dissolved in tetrahydrofuran (50 mg mL^{-1}) and applied by shortly immersing the capillary in the solution that is kept in the tip of a Pasteur pipette and left to cure overnight. The membrane was applied under microscopic guidance. The membrane separated the electrolyte from the seawater but was permeable for hydrogen peroxide. After mounting the electrodes in the casing, the sensor was filled with electrolyte, a borate/potassium chloride buffer (50 mM borate, 3 M potassium chloride and 500 µM ferrozine), with pH 9.

The selectivity was assessed by addition of potentially interfering compounds (ammonium, nitrate, nitrite, formate, acetic acid, ascorbate, and Fe^{2+} (Fe^{2+} additions tested at pH 3)). The response of the sensor was assessed for incremental additions of these compounds, and for hydrogen peroxide (3% hydrogen peroxide stock solution). Sensitivity was defined as the slope of the linear trend line. The sensitivity for hydrogen peroxide was 9×10^{-5} pA μM^{-1}. Interference (in %) was calculated as: sensitivity interfering compound/sensitivity $H_2O_2 \times 100$ (Supplementary Table 7; Supplementary Fig. 12).

When no ferrozine was added to the electrolyte, the sensor had strong interference with Fe^{2+} (Supplementary Fig. 12). Sensors with ferrozine in the electrolyte had no response to Fe^{2+}

(Supplementary Fig. 2). The sensitivity for Fe^{2+} of a sensor with ferrozine (50 μM) was tested by step-wise addition of Fe^{2+} to 500 mL of N_2-flushed seawater of pH 3. Fe^{2+} was tested in final concentrations of 20, 100, and 200 μM, which covers the range of Fe^{2+} concentrations measured (Supplementary Fig. 3), and the signal of the sensor was recorded over time. Ferrozine-filled sensors (with a ferrozine concentration of 500 μM) were used for the measurements of this study. The sensor was connected to a picoammeter and polarized at +700 mV until reaching a stable current, which happened normally within an hour. The medium in which the sensor was used was connected to an external reference electrode. The sensors were calibrated before use in a stirred beaker with filtered seawater to which aliquots of stabilized 3% hydrogen peroxide were added. The response times were <3 seconds. The sensor was very stable and not noise sensitive. The sensitivity was 2×10^{-4} pA μM^{-1} (Supplementary Fig. 13). The response to hydrogen peroxide was linear within the range of 0.5 μM to 4.3 mM (Supplementary Fig. 13), the highest concentration tested. The sensor was slightly light-sensitive. The shelf lifetime of the sensor was a few weeks. With use in anoxic sediments the sensitivity quickly goes down after a while.

Supplementary References

1 Tjell, A. Ø. & Almdal, K. Diffusion rate of hydrogen peroxide through water-swelled polyurethane membranes. *Sens. Bio-Sens. Res.* **21**, 35-39 (2018).
